# PLUTO: a YOLO-based lung field detector for pediatric lateral chest X-rays generalizable to adults

**DOI:** 10.3389/frai.2026.1874287

**Published:** 2026-07-20

**Authors:** Sivaramakrishnan Rajaraman, Renee Browning, Patrick Jean-Philippe, Carlos M. Perez-Velez, Sameer Antani

**Affiliations:** 1 Division of Intramural Research, National Library of Medicine, National Institutes of Health, Bethesda, MD, United States; 2 National Institute of Allergy and Infectious Diseases, National Institutes of Health, Bethesda, MD, United States; 3 Division of Infectious Diseases, School of Medicine, University of New Mexico, Albuquerque, NM, United States

**Keywords:** adult generalizability, deep learning, lateral chest X-rays, lung field detection, pediatric, YOLO11 object detection

## Abstract

**Introduction:**

Lateral chest X-rays (CXRs) are very important for detecting tuberculosis (TB) in infants and children, particularly for assessing TB-related lymphadenopathy and intrathoracic structures that are obscured in frontal projections. Although deep learning (DL)–based artificial intelligence (AI) has advanced CXR analysis, lateral projection imaging remains largely unexplored. Lung field detection is a critical first step in such pipelines, enabling DL models to focus on the relevant anatomy and improving downstream tasks such as disease detection, classification, and clinical decision support.

**Methods:**

Our DL-based model, called Pediatric Lateral lUng deTection with yOlo (PLUTO), enables lateral lung field detection not only in pediatric CXRs, but also demonstrates cross-domain generalizability to adult lateral CXRs. Related to prior work in this emerging area, PLUTO advances the field through a systematic age-stratified evaluation framework and an explicit assessment of cross-domain transfer to adult images. PLUTO uses a YOLO11s detector backbone selected after evaluating multiple state-of-the-art YOLO11 variants through five-fold cross-validation on age-stratified pediatric CXRs. The test cohort includes internal pediatric hold-out data and external pediatric and adult CXRs.

**Results:**

PLUTO achieved strong performance, with mAP@[0.5:0.95] scores of 0.8816 ± 0.0061 (internal pediatric) and 0.8898 ± 0.0084 (external pediatric), and demonstrated preliminary cross-domain generalizability to adult images. However, confirmation at a larger scale remains an important direction for future work. PLUTO also improved zero-shot lateral lung field segmentation performance.

**Discussion:**

The PLUTO model provides a valuable resource for anatomically grounded AI in lateral pediatric TB imaging and will enhance research in pulmonary TB and related diseases.

## 1 Introduction

Chest imaging is a standard component of the initial diagnostic evaluation for pediatric tuberculosis (TB) and other intrathoracic diseases, with chest X-ray (CXR) being the most commonly used first-line imaging modality (Wang et al., 2021). CXR imaging typically includes a frontal projection, with an additional lateral projection obtained less frequently (Perez-Velez and Marais, 2012); however, the lateral view provides complementary diagnostic information that is not reliably obtainable from the frontal view alone, particularly for mediastinal lymphadenopathy, retrosternal and retrocardiac pathology, and posterior lower-lobe disease, and these features are especially relevant in childhood and adolescent tuberculosis and pneumonia (Tsitsiou et al., 2022; Hernanz-Lobo et al., 2025). In children, a defining radiographic feature of primary tuberculosis is intrathoracic lymphadenopathy, often hilar or mediastinal, which may be occult or poorly characterized on frontal projections but more readily appreciated on lateral views. For this reason, the World Health Organization (WHO) recommends that the CXR evaluation of children suspected of having intrathoracic TB include both frontal and lateral views (World Health Organization, 2021). Recent evidence further strengthens the case for including lateral radiographs in pediatric TB evaluation. A 2025 multi-site study demonstrated that adding lateral views significantly improved diagnostic performance across both high- and low-burden settings, increasing sensitivity for confirmed TB from 39.1 to 53.6% compared with frontal views alone (Hernanz-Lobo et al., 2025). Lateral radiographs have also been shown to improve the detection of abnormalities that may be missed on frontal images alone, particularly in non-endemic regions, where TB prevalence is low and clinical suspicion may be limited (Lee et al., 2011). These findings underscore that lateral imaging is not merely optional but can meaningfully enhance TB detection, especially in children whose radiographic findings are subtle or atypical.

Despite these advantages, most publicly available pediatric CXR datasets lack lateral projections (Kermany et al., 2018; Nguyen et al., 2023). High-quality lateral imaging is also technically challenging in young children due to motion, positioning difficulties, and limited cooperation (Thukral, 2015). While lateral projections constitute a meaningful proportion of several major adult CXR repositories, approximately 31–32% of MIMIC-CXR and PadChest images, approximately 15% of CheXpert, and over 25% of BRAX, these lateral views remain substantially underutilized in DL research, and no publicly available collection provides sufficient annotated pediatric lateral CXRs to support systematic AI model development in this population (Bustos et al., 2020; Johnson et al., 2019; Wang et al., 2019; Nguyen et al., 2022; Reis et al., 2022). As a result, while deep learning (DL) methods have advanced rapidly for adult frontal CXRs, progress in pediatric lateral CXR analysis has been minimal.

This study addresses gaps in prior work that has demonstrated the feasibility of DL-based lung field localization in lateral pediatric CXRs (Capellán-Martín et al., 2023, 2025). Our work improves upon these through a systematically validated approach that explicitly accounts for age-dependent anatomical variability across the full spectrum of pediatric development, with cross-domain assessment of generalizability to adults. Lung field localization is a critical prerequisite for downstream disease-detection tasks because the pediatric thorax differs substantially from the adult thorax and undergoes dramatic developmental changes from infancy through adolescence. These changes include shifts in thoracic geometry, lung volume, rib orientation, and parenchymal distribution (Perez-Velez and Marais, 2012). Such anatomical variability introduces substantial heterogeneity in appearance, making consistent visual interpretation difficult and limiting the generalizability of DL models trained on adult or frontal-only datasets (Candemir and Antani, 2019). Accurate localization of the pulmonary region of interest (RoI) provides a necessary spatial prior for AI-based disease detection. Lung field detection improves downstream diagnostic performance, enhances reliability, and reduces computational burden by focusing the diagnostic model on relevant lung structure and excluding non-pulmonary anatomy.

## 2 Related work

Lateral lung field detection in pediatric CXRs. The closest antecedent to our Pediatric Lateral lUng deTection with yOlo model, which we call PLUTO, that detects lung fields in pediatric lateral CXRs, is a work on pediatric TB detection (Capellán-Martín et al., 2023), which demonstrated the initial feasibility of DL-based lateral lung field detection in pediatric chest radiographs. That study established the viability of automating this task and provided a foundational reference for the approach adopted here. Building upon this foundation, PLUTO advances the field by introducing age-stratified evaluation across four developmental cohorts, a systematic comparison of YOLO11 architecture variants, cross-domain assessment of adult generalizability, and downstream integration with a foundation segmentation model. The clinical importance of lateral CXR analysis in pediatric TB is further evidenced by a recent multi-view deep learning study (Capellán-Martín et al., 2025), which employed lateral lung ROI extraction, using a pipeline analogous to PLUTO’s, as a foundational preprocessing step within a system that jointly analyzes frontal and lateral CXR projections to improve pediatric TB classification. That study demonstrated that incorporating lateral views meaningfully improves diagnostic accuracy, directly motivating the need for reliable, automated lateral lung field localization of the kind PLUTO provides.

The value of jointly processing frontal and lateral CXR projections has been demonstrated for adult imaging as well. DualNet (Rubin et al., 2018) introduced an architecture that simultaneously processes a patient’s frontal and lateral CXRs, demonstrating improved detection performance across multiple radiological findings in predominantly adult data relative to single-view baselines. This line of work underscores the diagnostic complementarity of the two projections and motivates the development of automated lateral lung field localization as a prerequisite for such multi-view systems in the pediatric domain. MVC-Net (Zhu and Feng, 2021) extended the multi-view concept by proposing a classification network that fuses paired frontal and lateral CXR representations at both the feature and decision levels. While developed primarily for adult data, MVC-Net demonstrates the architectural patterns through which lateral view information can be integrated into diagnostic pipelines. Together, these prior studies establish a clear trajectory: lateral CXR analysis provides complementary diagnostic information beyond what frontal projections alone can offer, multi-view fusion architectures can exploit this complementarity for improved disease detection, and reliable automated lateral lung field localization is a prerequisite for building such pipelines in the pediatric domain. PLUTO addresses this prerequisite systematically.

Therefore, as a first step in AI-driven disease detection, we need to localize the pulmonary RoI. Precise detection of the lung field serves as a spatial prior, focusing a diagnostic model’s attention on relevant structures and minimizing the influence of non-pulmonary anatomy, thereby improving performance, reliability, and computational efficiency, as demonstrated in prior RoI-guided medical imaging pipelines. This manuscript proposes a DL-based lateral lung field detection model specifically trained on pediatric CXRs that is evaluated systematically across age groups and external cohorts. This strategy serves as a foundational step toward automated pediatric TB detection and broader analysis of pediatric thoracic disease. For evaluation, we use pediatric CXRs from the National Institute of Allergy and Infectious Diseases (NIAID) AI research study (NIAID pediatric-CXR dataset), enabling rigorous assessment and supporting future research in pediatric and cross-domain analysis.

## 3 Algorithmic workflow

**Figure 1:**
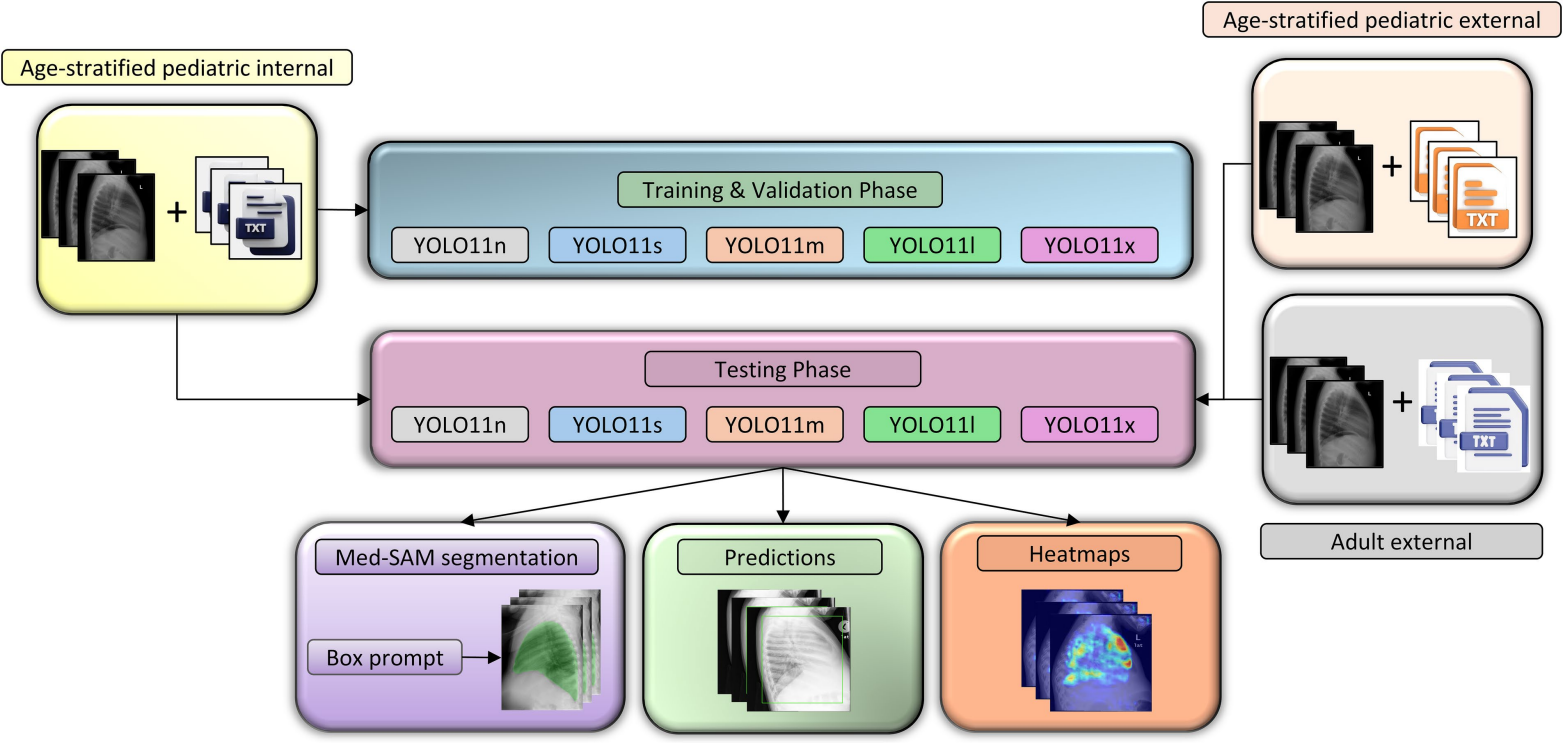
Schematic overview of the PLUTO pipeline for lung field detection in lateral chest radiographs. Pediatric lateral CXRs are age-stratified and used to train and evaluate YOLO11 model variants via five-fold cross-validation. The best-performing variant (YOLO11s) is designated PLUTO and evaluated on internal pediatric hold-out data, external pediatric CXRs (PadChest), and external adult CXRs (PadChest and IU CXR) to assess cross-domain generalizability. Statistical significance of inter-variant performance differences is assessed using the Wilcoxon signed-rank test. Outputs include lung bounding box predictions, activation heatmaps, and MedSAM-based zero-shot segmentation guided by bounding box detections. Representative real lateral CXR images are shown; all images are de-identified.

The contribution of our study is a systematically evaluated lateral lung field detection algorithm based on the YOLO11s detector (Khanam and Hussain, 2024) that detects lung fields in pediatric lateral CXRs and demonstrates preliminary cross-domain generalizability to adult lateral CXRs, building upon prior work in this emerging area (Capellán-Martín et al., 2023). An overview of the study design and methodology is presented in Figure 1.

The principal contributions of this work, building upon and extending prior research (Capellán-Martín et al., 2023), are detailed through the following systematic procedure:

- We implemented an age-stratified evaluation strategy across four clinically relevant developmental cohorts (0 years to < 2 years, 2 years to < 5 years, 5 years to < 11 years, and 11 years to < 18 years, respectively), thereby addressing the anatomical variability inherent in pediatric populations.
- All YOLO11 variants were systematically evaluated to identify the best-performing model, with internal NIAID pediatric-CXR data and external hold-out testing on the pediatric data from the publicly available PadChest CXR dataset (Bustos et al., 2020).
- We assessed PLUTO’s generalizability to domain shift by also evaluating it with adult lateral CXRs from two publicly available external sources, viz., PadChest and Indiana University (IU) (Demner-Fushman et al., 2016) CXR collections, respectively.
- We used the Wilcoxon signed-rank test (Rosner et al., 2006) to assess the statistical significance of performance differences across YOLO11 variants during both internal and external validation to demonstrate the consistency of our findings.
- We evaluated the benefits of spatially targeted lateral lung field detection on downstream zero-shot lateral lung segmentation using the Medical Segment Anything Model (MedSAM) (Ma et al., 2024).

## 4 Materials and methods

### 4.1 Data collection

This retrospective study used the following CXR datasets:

- NIAID pediatric-CXR: This multicenter international dataset was acquired by the National Institute of Allergy and Infectious Diseases (NIAID) aimed at innovating AI-driven approaches for TB detection in children with and without HIV. The images were acquired from South Africa, China, Peru, Bangladesh, Brazil, and Haiti. The study used existing, de-identified data that cannot be linked back to individual cases, and informed consent was not required. The de-identified data included a collection of imaging and case report forms for (i) microbiologically confirmed pediatric pulmonary TB cases (*n* = 721), (ii) unconfirmed TB (clinically diagnosed) cases (*n* = 81), and (iii) normal cases (controls) (*n* = 200). There were 543 patient-unique lateral CXR projections, which were further stratified into four age groups: 0 years to < 2 years (*n* = 225), 2 years to < 5 years (*n* = 159), 5 years to < 11 years (*n* = 150), and 11 years to < 18 years (*n* = 9). Lateral CXRs in the 11 years to < 18 years age group were underrepresented (*n* = 9), consistent with known acquisition patterns in pediatric imaging; while included to maintain age-stratified evaluation, findings in this subgroup should be interpreted qualitatively rather than as definitive age-specific performance estimates.
- PadChest CXR (Bustos et al., 2020): This publicly available de-identified CXR collection comprised over 160,000 adult and pediatric CXR images, both frontal and lateral, derived from routine clinical imaging and therefore representing a retrospective hospital-based convenience sample. Of these, only 23 were pediatric lateral CXRs with the following age distribution: 0 years to < 2 years (*n* = 4), 2 years to < 5 years (*n* = 9), 5 years to < 11 years (*n* = 6), and 11 years to < 18 years (*n* = 4). We used this pediatric set for external validation.
- Indiana University (IU) CXR (Demner-Fushman et al., 2016): This NLM/NIH-provided de-identified public dataset comprises 8,121 adult frontal and lateral CXRs and 3,996 associated radiology reports. There are 3,946 lateral CXR projections in this collection.
- A subset of adult lateral CXRs from the PadChest CXR (*n* = 100) and IU CXR (*n* = 100) collections, respectively, was individually used to test the cross-domain generalizability of the pediatric-trained models. Each image was manually annotated using a bounding box that encompasses the lung, and these bounding boxes were further verified by a board-certified clinical expert. This step helped evaluate whether a model trained with pediatric data could be adapted to the adult lung morphology.

The CXR data distribution for these datasets is shown in Table 1.

**Table 1:** Age-stratified data distribution of CXR data (Y = years).

Data	0Y - < 2Y	2Y - < 5Y	5Y - < 11Y	11Y - < 18Y	≥ 18Y (adults)	Total
NIAID pediatric-CXR	225	159	150	9	0	543
PadChest pediatric CXR	4	9	6	4	0	23
PadChest adult CXR	—	—	—	—	100	100
IU CXR	—	—	—	—	100	100

The distribution of the NIAID pediatric-CXR dataset across the development and test sets is shown in Table 2. For a comprehensive evaluation of the models’ performance, we performed five-fold cross-validation using the development set; the number of age-stratified samples in each fold is shown in Table 3. Cross-validation folds were constructed at the patient level, ensuring that no individual contributed images to more than one fold.

**Table 2:** Dataset partitioning of the NIAID pediatric-CXR data into development and test sets, by age group (Y = years).

Age group	Development (80%)	Test (20%)
0Y – < 2Y	180	45
2Y – < 5Y	127	32
5Y – < 11Y	120	30
11Y – < 18Y	7	2
Total	434	109

The development set is used for five-fold cross-validation; the test set is held out for internal evaluation only.

**Table 3:** NIAID pediatric-CXR development-set split for five-fold cross-validation, by age group (Y = years).

Age group	F1-T	F1-V	F2-T	F2-V	F3-T	F3-V	F4-T	F4-V	F5-T	F5-V
0Y – < 2Y	144	36	144	36	144	36	144	36	144	36
2Y – < 5Y	101	26	101	26	102	25	102	25	102	25
5Y – < 11Y	96	24	96	24	96	24	96	24	96	24
11Y – < 18Y	5	2	5	2	6	1	6	1	6	1

Each fold contributes a patient-disjoint validation subset, with the remaining four folds used for training. F1, F2, F3, F4, F5 denote the first, second, third, fourth, and fifth fold, respectively. T and V denote the train and validation splits, respectively, within each fold.

### 4.2 Lung field annotation

**Figure 2:**
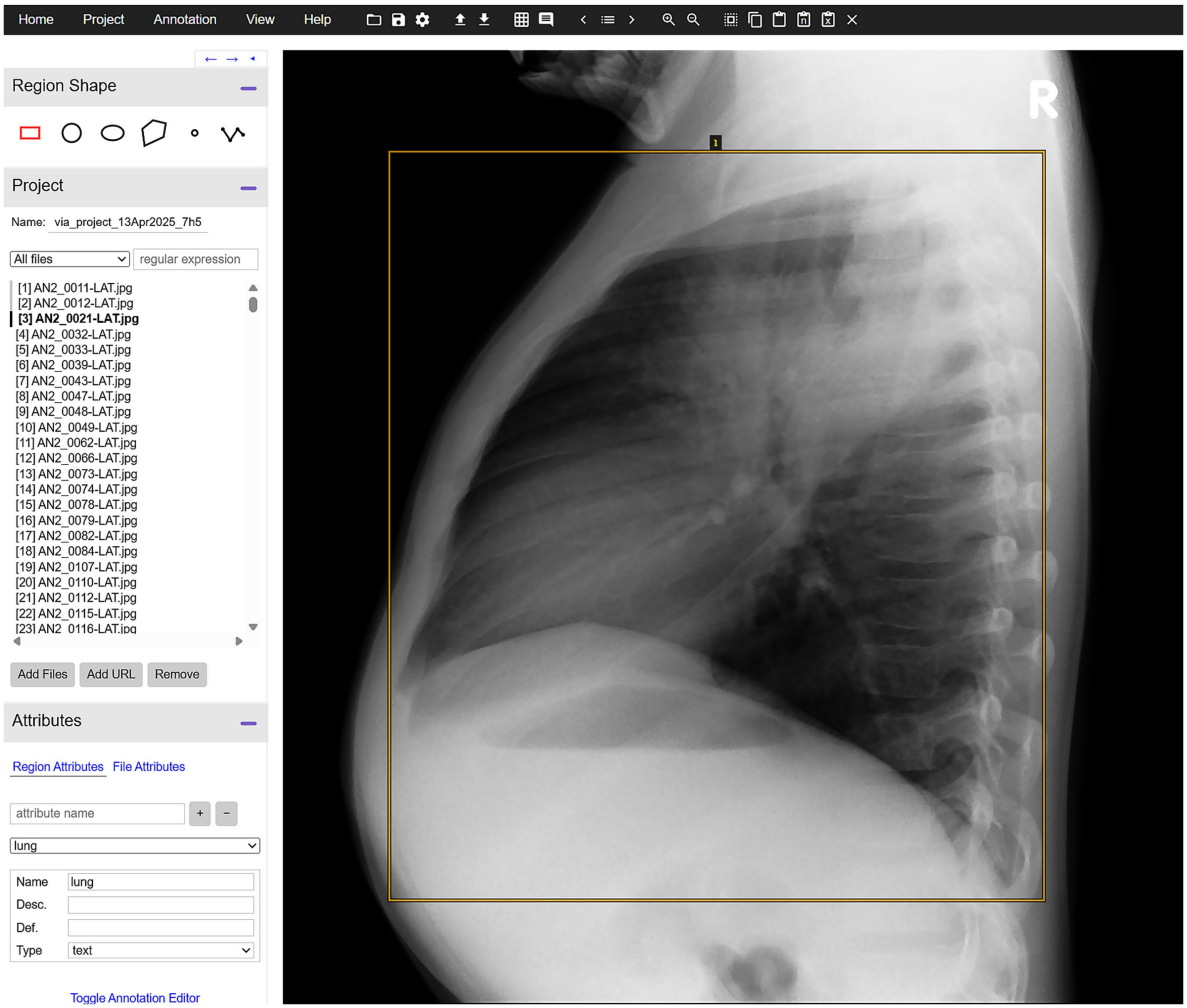
Annotating the lateral lung field using VGG Image Annotator (VIA). Interface overview, project configuration, and sample rectangular bounding box labeling.

We used the VGG Image Annotator (VIA) (Dutta and Zisserman, 2019) to manually annotate the lateral lung field with a rectangular bounding box. Rectangular bounding boxes were used to ensure compatibility with YOLO-based object detection frameworks and to provide a consistent spatial prior for lung field localization, which was sufficient for the study’s objective of RoI detection rather than fine-grained boundary delineation. Figure 2 shows VIA’s web-based interface with sample project files and a lateral CXR annotated with a rectangular bounding box defining the lung field. The box was carefully extended from the lung apex to the posterior costophrenic angle, ensuring complete coverage of the retrosternal airspace and lateral lung field. A board-certified infectious pulmonary disease clinician with over 20 years of experience in pediatric thoracic imaging independently reviewed and verified all annotations, confirming anatomical correctness and requesting corrections where necessary. This two-stage process, primary annotation by a trained annotator followed by independent expert verification, constitutes a structured quality assurance protocol. The relatively low ambiguity of rectangular bounding box annotation for a gross anatomical region such as the lateral lung field, defined by consistent landmarks including the lung apex and posterior costophrenic angle, mitigates but does not eliminate the risk of annotation bias. Multi-annotator designs with formal agreement analyses represent an important methodological improvement for future work in this area.

### 4.3 YOLO-compatible label creation

**Figure 3:**
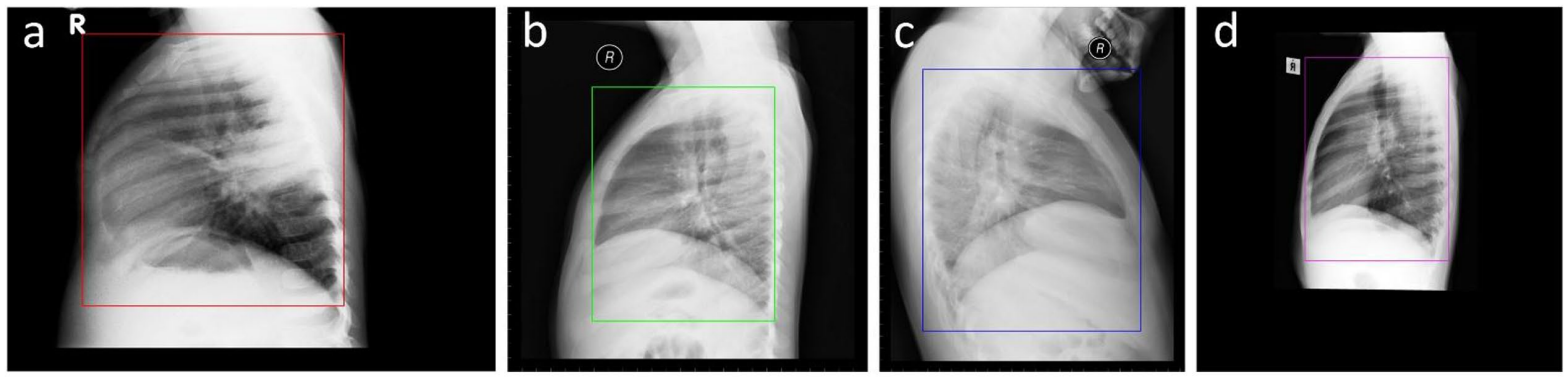
Representative pediatric lateral CXRs with age-stratified lung field annotations. Bounding boxes are color-coded by age group: **(a)** 0 years to < 2 years (red), **(b)** 2 years to < 5 years (green), **(c)** 5 years to < 11 years (blue), and **(d)** 11 years to < 18 years (cyan).

Label files were generated in YOLO-compatible structured text file format, where each row in the file corresponded to a rectangular bounding box definition using the following parameters, viz., [class x-center y-center width height]. The class number is zero-indexed (0: Lung). Coordinate values were normalized within a 0–1 range by dividing measurements by the image dimensions. The annotated images and their corresponding label files were used for YOLO training, validation, and subsequent testing. Figure 3 presents sample CXRs from different age groups where the lateral lung field is annotated with the bounding box.

### 4.4 Model architecture and loss function

**Figure 4:**
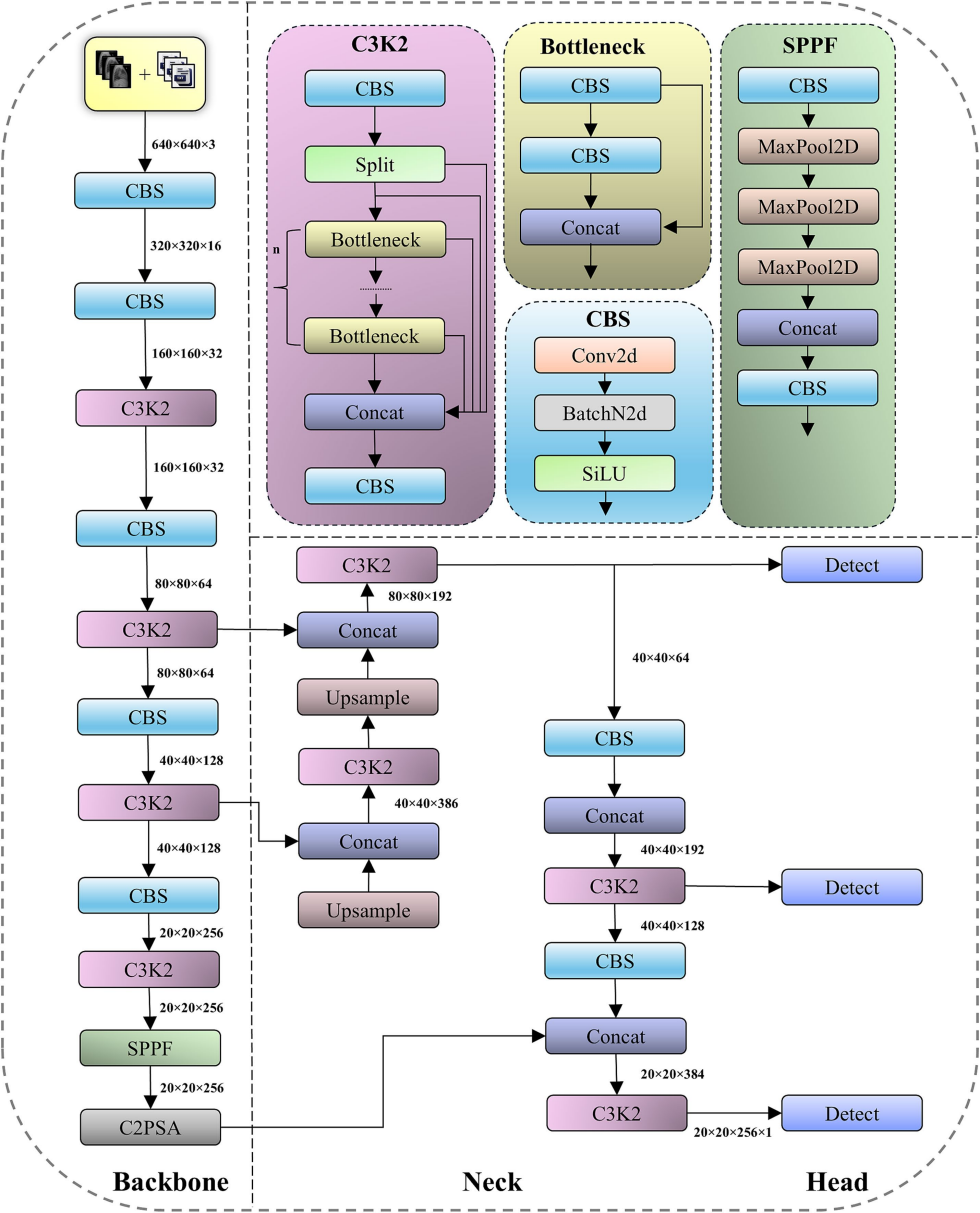
YOLO11 basic architecture. In the figure above, CBS are the convolutional blocks, SiLU denotes sigmoid linear unit activation, C3K2 denotes the Cross Stage Partial Bottleneck with 3 × 3 Kernels, SPPF denotes the Spatial Pyramid Pooling Fast module, Concat denotes channel concatenation that performs feature fusion, and C2PSA denotes the Cross Stage Partial with Spatial Attention block, respectively.

This work used an unmodified YOLO11 architecture (Khanam and Hussain, 2024) from Ultralytics^®^, comprising the backbone, neck, and output layers, as shown in Figure 4. The model is available in multiple variants, YOLO11n, YOLO11s, YOLO11m, YOLO11l, and YOLO11x, respectively, each designed to balance network complexity and inference speed. The YOLO11 model is trained by minimizing a composite loss function $L_{\text{YOLO}11}$ that comprises three terms, each targeting a distinct aspect of detection quality: $L_{\text{class}}$ penalizes errors in class probability prediction, $L_{\mathrm{box}}$ penalizes geometric misalignment between predicted and ground-truth bounding boxes, and $L_{\mathrm{dfl}}$ regularizes bounding box coordinate regression by modeling coordinate uncertainty as a learned probability distribution.


$$L_{\text{YOLO}11}=L_{\text{class}}+L_{\mathrm{box}}+L_{\mathrm{dfl}}$$

The classification loss $L_{\text{class}}$ measures the discrepancy between predicted class probabilities and ground-truth labels using cross-entropy:


$$L_{\text{class}}=-\frac{1}{N}\sum_{i=1}^{N}[y_{i}\;\log(\sigma (p_{i}))+(1-y_{i})\;\log(1-\sigma (p_{i}))]$$

Here, $y_{i}$ is the ground-truth label for the $i^{\mathrm{th}}$ prediction, $p_{i}$ is the predicted probability, and N is the total number of predictions. The bounding-box regression loss $L_{\mathrm{box}}$ quantifies how accurately the predicted bounding boxes align with the ground truth. This alignment is measured using Complete Intersection over Union (CIoU), a metric that extends standard IoU by subtracting two additional penalty terms to account for distance and shape. The distance penalty is calculated as the squared Euclidean distance between the center points of the predicted and ground-truth boxes, normalized by the diagonal length of the smallest enclosing box. The aspect ratio penalty measures the difference in the width-to-height ratio between the boxes, typically using an arctangent-based function.

A CIoU of 1 indicates perfect alignment, whereas lower values reflect increasing spatial discrepancy:


$$L_{\mathrm{box}}=\frac{1}{N_{\text{bbox}}}\sum_{i=1}^{N_{\text{bbox}}}[1-C\mathrm{IoU}(B_{i}^{\text{pred}},B_{i}^{\text{true}})]$$

Here, $B_{i}^{\text{pred}}$ and $B_{i}^{\text{true}}$ denote the predicted and ground-truth bounding boxes for the $i^{\mathrm{th}}$ object. While CIoU is utilized as the regression loss to optimize bounding box convergence via center distance and aspect ratio penalties, standard IoU is strictly employed as the evaluation metric to maintain consistency with global benchmarks like COCO for AP and mAP calculations.

The distribution focal loss $L_{\mathrm{dfl}}\;$ addresses bounding box coordinate regression by reformulating it as a classification problem over a discrete set of evenly spaced candidate values. The model learns a probability distribution over discrete bin positions rather than predicting a single real-valued coordinate offset. $L_{\mathrm{dfl}}\;$ minimizes a cross-entropy loss between the predicted distribution and the two adjacent discrete bins that bracket the true continuous coordinate value, thereby encouraging the predicted distribution to concentrate around the ground truth. Here, y is the ground-truth continuous bounding box coordinate value, $y_{i}$ and $y_{i+1}$ are the two adjacent discrete bin positions satisfying $y_{i}$ ≤ y < $y_{i+1}$ , and $p_{i}$ and $p_{i+1}$ are the corresponding predicted probabilities. This formulation produces stable training gradients and enables the model to represent uncertainty in box coordinate prediction.


$$L_{\mathrm{dfl}}=-((y_{i+1}-y)\log(p_{i})+(y-y_{i})\log(p_{i+1}))$$

### 4.5 Evaluation metric

Model performance was evaluated using the mean Average Precision (mAP@[0.5:0.95]) metric, the standard benchmark for object-detection tasks (Khanam and Hussain, 2024). This metric is derived from Average Precision (AP), which summarizes detection quality as the area under the precision–recall (PR) curve for a given IoU threshold. Since AP is computed at multiple IoU thresholds, mAP inherently captures the model’s bounding-box accuracy across a range of localization strictness levels. For a given IoU threshold t, AP is computed using the Ultralytics YOLO evaluation module, which interpolates the PR curve at 101 uniformly spaced recall points:


$$\mathrm{AP}(t)\approx \frac{1}{101}\;\sum_{i=1}^{101}p(r_{i})$$

The mAP@[0.5:0.95] score is obtained by averaging the AP values across T = 10 distinct IoU thresholds ranging from 0.50 to 0.95 in increments of 0.05:


$$\mathrm{mAP}@[0.5:0.95]=\frac{1}{T}\sum_{t=1}^{T}\mathrm{AP}(t),\text{with}\;T=10$$

Since our task involves a single object class (the lateral lung), the per-class AP and the dataset-level mAP are equivalent at any individual threshold. We report the mean and standard deviation of the mAP@[0.5:0.95] values across cross-validation folds to summarize overall performance with *K* = 5 folds. This formulation ensures that both localization accuracy (via IoU) and detection confidence (via precision–recall behavior) are jointly reflected in the final performance metric:


$$\mathrm{mAP}@[0.5:0.95]=1/K\Sigma_{\left(i=1\right)}^{\left(K\right)}\mathrm{mAP}@{\left[0.5:0.95\right]}_{i}$$

$$\sigma_{{\mathrm{mAP}}_{\left[.5:.95\right]}}=\sqrt{\frac{1}{K}\sum_{i=1}^{K}{\left({\mathrm{mAP}}_{[0.5:0.95],i}-{\bar{\mathrm{mAP}}}_{\left[0.5:0.95\right]}\right)}^{2}}$$

### 4.6 Training parameters and environment

The models were trained, validated, and tested using the Python 3.9 environment with Pytorch. Experiments were conducted on Amazon AWS SageMaker, using a single NVIDIA A10 GPU (24GB memory). Training parameters were set to the Ultralytics’ default settings, including using an AdamW optimizer with an initial learning rate of 0.001 and a momentum of 0.9. We used a batch size of 16, and the models were trained for 500 epochs. Early stopping was applied with patience (*n* = 100), while warmup epochs (*n* = 5) were used to facilitate stable convergence. A patience of 100 epochs was selected to accommodate the relatively small pediatric training set and the shallow, noisy loss landscape that can characterize training on anatomically variable pediatric data; lower patience values risk premature termination before the model has fully learned age-specific lung morphology patterns, particularly in the youngest and most morphologically distinctive age groups. Default spatial augmentations included translation and scaling to enhance regularization.

Model interpretability was assessed using EigenCAM (Bany Muhammad and Yeasin, 2021), which is a gradient-free class activation mapping method adapted for YOLO architectures. EigenCAM computes the first principal component of the spatial activation maps at a designated target layer via singular value decomposition, producing spatially continuous heatmap overlays that visualize the dominant feature representations learned by the model. The resulting map is resized to the input resolution and min-max normalized to [0, 1] before overlay. EigenCAM was applied at the Convolutional block with Partial Spatial Attention (C2PSA), the final semantic feature extraction layer before the YOLO11s detection head. Since PLUTO is a single-class detector, the dominant activation principal component at this layer corresponds anatomically to the lung field, providing interpretable spatial visualization of the model’s learned representations.

### 4.7 Statistical analysis

We used the Wilcoxon signed-rank test, a non-parametric method for comparing paired observations, to assess the statistical significance of performance variations across the model variants. The null hypothesis assumes no significant difference (*p* ≥ 0.05) in mAP@[0.5:0.95] between model pairs, while the alternative hypothesis suggests a significant difference (*p* < 0.05). The test statistic W is computed as,


$$W=\sum_{i=1}^{n}\mathrm{sgn}(d_{i})\cdot R_{i}$$

Here, n denotes the number of paired observations, $d_{i}$ denotes the difference in mAP@[0.5:0.95] between the paired observations for the $i^{\mathrm{th}}$ instance, $\mathrm{sgn}(d_{i})$ denotes the sign of $d_{i}$ , i.e., +1 if $\left(d_{i}>0\right)$ , −1 if $\left(d_{i}<0\right)$ , and $R_{i}$ denotes the rank of the absolute value $|d_{i}|$ .

## 5 Results

Table 4 shows the performance of all YOLO11 variants, expressed as mean and standard deviations of the mAP@[0.5:0.95]. The internal test performance indicated that YOLO11s achieved the numerically highest mAP@[0.5:0.95] (0.8816 ± 0.0061), closely followed by YOLO11l and YOLO11m, while YOLO11n and YOLO11x had slightly lower scores. YOLO11s also achieved the highest mAP@[0.5:0.95] (0.8898 ± 0.0084) on the external pediatric test, with YOLO11m performing competitively. YOLO11s showed a consistent pattern of numerical leadership across both internal and external pediatric test sets, although these differences did not reach statistical significance (*p* > 0.05, Wilcoxon signed-rank test), indicating practically equivalent performance across YOLO11 variants on this task.

**Table 4:** Internal and external pediatric test performance.

Model	Int-mAP@[0.5:0.95]	Ext-mAP@[0.5:0.95]
YOLO11n	0.8686 ± 0.0059	0.8692 ± 0.0142
YOLO11s	**0.8816 ± 0.0061**	**0.8898 ± 0.0084**
YOLO11m	0.8808 ± 0.0052	0.8834 ± 0.0127
YOLO11l	**0.8816 ± 0.0061**	0.8652 ± 0.0123
YOLO11x	0.8776 ± 0.0094	0.8718 ± 0.0245

Int and Ext denote the internal NIAID pediatric-CXR (*n* = 109) and external PadChest pediatric (*N* = 23) test performance, respectively. Bold numerical values within each column denote the numerically highest performance. The internal and external columns differ in sample size, dataset origin, and acquisition characteristics; direct cross-column comparison is not intended.

The age-stratified performance with the internal NIAID pediatric-CXR test is reported in Table 5. YOLO11s showed the highest mAP@[0.5:0.95] across most pediatric groups, except for ages 5 to <11 years, where YOLO11m had a slightly higher score (0.8772 ± 0.0092 vs. 0.8742 ± 0.0100). Results for ages 11 to <18 years should be viewed with caution due to the small sample size (*n* = 9).

**Table 5:** Age-stratified internal NIAID pediatric-CXR test performance (total *N* = 109; 0Y–<2Y: *n* = 45, 2Y–<5Y: *n* = 32, 5Y–<11Y: *n* = 30, 11Y–<18Y: *n* = 2).

Model	Int-mAP@[0.5:0.95]			
	0Y - < 2Y	2Y - < 5Y	5Y - < 11Y	11Y - < 18Y
YOLO11n	0.8614 ± 0.0083	0.886 ± 0.0147	0.8542 ± 0.0147	0.9136 ± 0.0234
YOLO11s	**0.876 ± 0.0050**	**0.9002 ± 0.0101**	0.8742 ± 0.0100	**0.9744 ± 0.0261**
YOLO11m	0.8700 ± 0.0062	0.8936 ± 0.0045	**0.8772 ± 0.0092**	0.9246 ± 0.0695
YOLO11l	0.8658 ± 0.0120	0.8944 ± 0.0054	0.8608 ± 0.0195	0.9652 ± 0.0270
YOLO11x	0.8688 ± 0.0062	0.8952 ± 0.0141	0.8662 ± 0.0146	0.9278 ± 0.0283

Bold numerical values within each column denote the numerically highest performance (Y = years). Results for the 11 years to <18 years sub-group (*n* = 9 in the full dataset; *n* = 2 in the test set) should be interpreted descriptively due to the very small sample size; quantitative conclusions for this age range are not supported.

The age-stratified performance with the external PadChest pediatric test cohort is reported in Table 6. Here, both YOLO11s and YOLO11m reported high performance. For the pediatric age group 0 years to < 2 years and 5 years to < 11 years, respectively, the YOLO11m outperformed all other models. Conversely, YOLO11s outperformed all other models for the age group 2 years to < 5 years and 11 years to < 18 years, respectively. In the non-age-stratified evaluation, the test compared aggregate mAP@[0.5:0.95] scores across all pediatric samples, while comparisons were performed within each age group in the age-stratified setting to determine whether differences between variants were statistically significant. Across all comparisons, the Wilcoxon signed-rank test yielded *p* > 0.05, indicating no statistically significant performance differences between any YOLO11 variant pair. This finding reflects the overall robustness of the YOLO11 architecture family for the lateral lung field detection task, suggesting that reliable performance is achievable across a range of model complexities. Despite the absence of statistically significant pairwise differences, YOLO11s showed a consistent pattern of numerical leadership across most subgroups and overall evaluations. Given this consistency, combined with YOLO11s’s favorable parameter efficiency relative to larger variants (YOLO11m, YOLO11l, YOLO11x), we selected YOLO11s as the PLUTO backbone, a choice guided by both numerical consistency and practical deployment considerations rather than statistical superiority.

**Table 6:** Age-stratified external PadChest pediatric test performance (total *N* = 23; 0Y–<2Y: *n* = 4, 2Y–<5Y: *n* = 9, 5Y–<11Y: *n* = 6, 11Y–<18Y: *n* = 4).

Model	Ext-mAP@[0.5:0.95]			
	0Y - < 2Y	2Y - < 5Y	5Y - < 11Y	11Y - < 18Y
YOLO11n	0.8310 ± 0.0287	0.8862 ± 0.0237	0.9050 ± 0.0200	0.8420 ± 0.0239
YOLO11s	0.8748 ± 0.0301	**0.9002 ± 0.0166**	0.9134 ± 0.0159	**0.8892 ± 0.0324**
YOLO11m	**0.8852 ± 0.0366**	0.8872 ± 0.0096	**0.9138 ± 0.0446**	0.8626 ± 0.0168
YOLO11l	0.8634 ± 0.0138	0.8788 ± 0.0177	0.8890 ± 0.0326	0.8448 ± 0.0341
YOLO11x	0.8506 ± 0.0493	0.8846 ± 0.0112	0.9136 ± 0.0375	0.8486 ± 0.0395

Bold numerical values within each column denote the numerically highest performance (Y = years). Per-subgroup sample sizes are small; values are reported for completeness and should be interpreted with appropriate caution.

**Figure 5:**
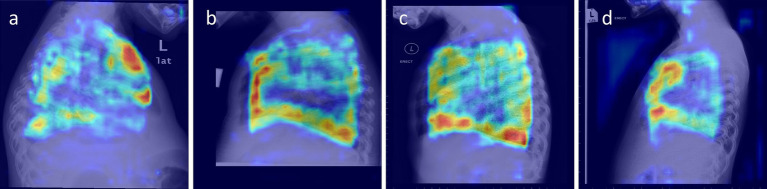
EigenCAM heatmaps overlaid on representative pediatric lateral CXRs from the internal pediatric test set. Heatmap colors indicate relative activation intensity within the spatial activation map, with warmer colors indicating stronger activation and cooler colors indicating weaker activation; red/orange regions represent the highest activation, yellow/green regions represent intermediate activation, and blue regions represent low activation. The overlays reflect the spatial distribution of the dominant activation principal component at the selected YOLO11s feature layer. Age-specific YOLO11s models were used for: **(a)** 0 years to <2 years, **(b)** 2 years to <5 years, **(c)** 5 years to <11 years, and **(d)** 11 years to <18 years.

Representative CXRs are shown in Figure 5, where EigenCAM heatmaps overlaid on the input images demonstrate spatially continuous activation patterns that align with the lung fields. This spatial correspondence between the dominant activation principal component and the lung region indicates that YOLO11s has learned to concentrate its internal representations on the lateral lung field across all pediatric age groups.

## 6 Discussion

### 6.1 Comparison with previous research

PLUTO improves upon a prior work that established the foundational feasibility of DL-based lateral lung field detection in pediatric CXRs (Capellán-Martín et al., 2023) in several specific directions. First, PLUTO follows a systematic age-stratified evaluation framework across four clinically defined developmental cohorts spanning birth through adolescence, explicitly addressing the anatomical variability inherent in pediatric development, which represents a distinct methodological advance over single-cohort or age-agnostic approaches. Second, the NIAID pediatric-CXR dataset used here is a multi-site international collection, providing demographic breadth beyond what single-center-sourced datasets offer. Third, PLUTO provides a comprehensive comparative evaluation of all five YOLO11 variants with non-parametric statistical significance testing, supporting transparent model selection. Fourth, PLUTO is explicitly evaluated for cross-domain adult generalizability on two independent external adult cohorts, a dimension not addressed in prior pediatric lateral detection work. Fifth, PLUTO’s detection output is evaluated as a guiding prior for downstream zero-shot segmentation using MedSAM, illustrating its utility within a broader AI pipeline. The downstream clinical relevance of this detection pipeline is further evidenced by recent work (Capellán-Martín et al., 2025), which employed a similar lateral lung ROI extraction strategy as a foundational component in a multi-view DL system for pediatric TB classification, achieving meaningful diagnostic improvements.

### 6.2 Training strategy and design rationale

The choice to train PLUTO exclusively on pediatric lateral CXRs and subsequently evaluate cross-domain transfer to adult images reflects a deliberate clinical and methodological rationale. The study’s primary objective is pediatric lateral lung field detection, and the pediatric thorax is anatomically distinct from the adult thorax in ways that are clinically significant. The rib orientation shifts from horizontal in infants to progressively oblique through childhood, lung volume and cardiac proportion change substantially across developmental stages, and diaphragm position varies with age (Perez-Velez and Marais, 2012). Training directly on pediatric data ensures that the model encodes these age-specific anatomical features rather than adult-biased representations that might reduce sensitivity in the youngest and most morphologically distinct age groups. Furthermore, evaluating transfer from the pediatric to the adult domain, rather than the reverse, constitutes a more conservative generalizability test wherein the model is asked to transfer from a smaller, more variable source domain to a larger, more anatomically homogeneous target domain. The encouraging cross-domain performance observed (mAP@[0.5:0.95] of 0.8100 ± 0.0203 on PadChest adult CXRs and 0.7890 ± 0.0183 on IU CXRs) suggests that PLUTO has learned meaningful features that are sufficiently general to transfer across this domain gap. Adult pre-training followed by pediatric fine-tuning represents an alternative strategy worth investigating in future work, and could be particularly relevant if larger annotated adult lateral CXR collections with consistent bounding box protocols become available. It is important to note that the selection of YOLO11s as the PLUTO backbone is not based on statistical superiority over other YOLO11 variants, as Wilcoxon signed-rank testing yielded *p* > 0.05 for all pairwise comparisons. Rather, the selection reflects YOLO11s’s consistent numerical leadership across evaluations combined with its favorable computational efficiency profile, a practically meaningful criterion for deployment in resource-constrained clinical environments.

### 6.3 Generalizability to adults

**Figure 6:**
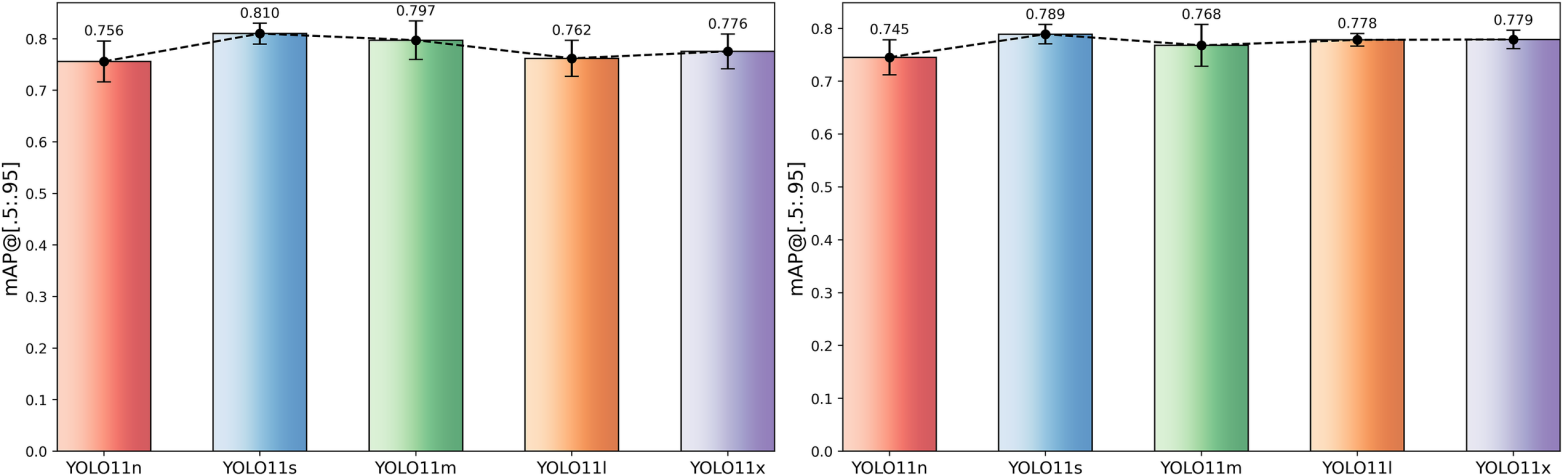
Bar plots illustrate the performance of pediatric-trained YOLO11 variants using adult lateral CXRs. Mean mAP@[0.5:0.95] scores with standard deviation are shown for the **(a)** PadChest and **(b)** IU CXR sets.

**Figure 7:**
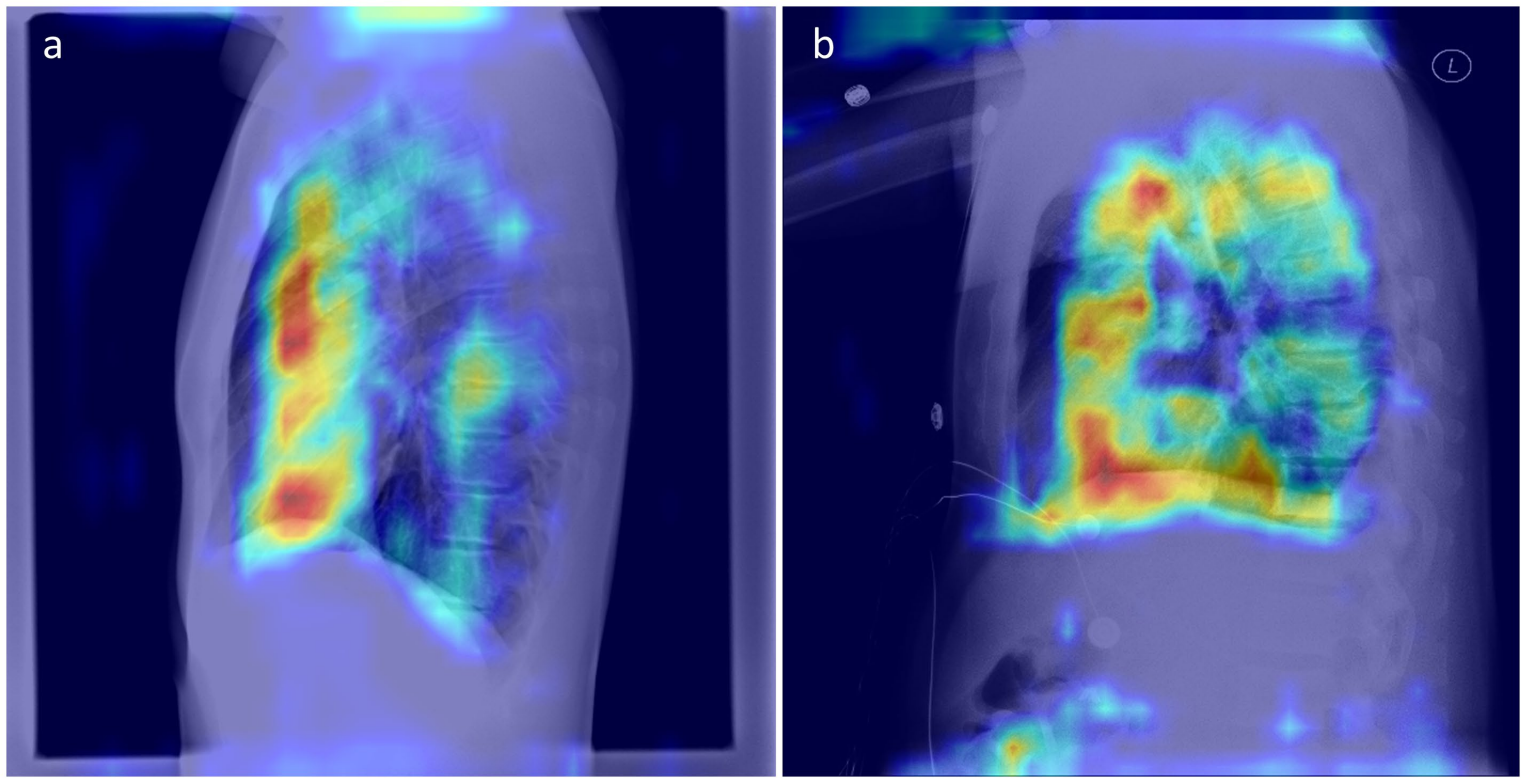
EigenCAM heatmaps overlaid on representative adult lateral CXRs from **(a)** PadChest and **(b)** IU CXR sets. Heatmap colors indicate relative activation intensity within the spatial activation map, with warmer colors indicating stronger activation and cooler colors indicating weaker activation; red/orange regions represent the highest activation, yellow/green regions represent intermediate activation, and blue regions represent low activation. The spatial alignment of high-activation regions with the lateral lung field confirms that PLUTO’s learned feature representations transfer to adult thoracic morphology.

The pediatric-trained models demonstrated encouraging preliminary cross-domain generalizability to adult lateral CXRs. As summarized in Table 7 and illustrated in Figures 6, 7, the pediatric-trained models consistently maintained high detection performance with the adult CXRs, where the YOLO11s model outperformed its counterparts, achieving mAP@[0.5:0.95] of 0.8100 ± 0.0203 on the PadChest adult CXR set and 0.7890 ± 0.0183 on the IU CXR set, respectively. While these scores were moderately lower than the internal (0.8816 ± 0.0061) and external pediatric (0.8898 ± 0.0084) test performances, the reduction was relatively contained given the substantial anatomical differences between pediatric and adult thoracic morphology. Nevertheless, given that each adult evaluation cohort comprised 100 images, these results should be interpreted as a preliminary cross-domain generalizability assessment rather than a definitive validation. Larger-scale adult evaluation and adult-specific fine-tuning represent important directions for future work.

**Table 7:** Cross-domain generalizability evaluation using external adult lateral CXRs.

Model	P-mAP@[0.5:0.95]	I-mAP@[0.5:0.95]
YOLO11n	0.7560 ± 0.0398	0.7452 ± 0.0333
YOLO11s	**0.8100 ± 0.0203**	**0.7890 ± 0.0183**
YOLO11m	0.7972 ± 0.0373	0.7678 ± 0.0395
YOLO11l	0.7620 ± 0.0351	0.7782 ± 0.0120
YOLO11x	0.7756 ± 0.0338	0.7790 ± 0.0173

Values show mean ± SD of mAP@[0.5:0.95] for the PadChest adult (P, *N* = 100) and IU CXR (I, *N* = 100) subsets, evaluated using models trained exclusively on pediatric data. Bold numerical values within each column denote the numerically highest performance. Results should be interpreted as preliminary cross-domain generalizability evidence rather than definitive performance benchmarks.

It is observed from Figure 7 that the YOLO11s model learned anatomically grounded feature representations that transferred across domains. Such generalizability implies the feasibility of a unified age-independent detection pipeline, reducing the operational complexity associated with maintaining separate pediatric and adult lung field detection systems.

### 6.4 Detection-guided segmentation

**Figure 8:**
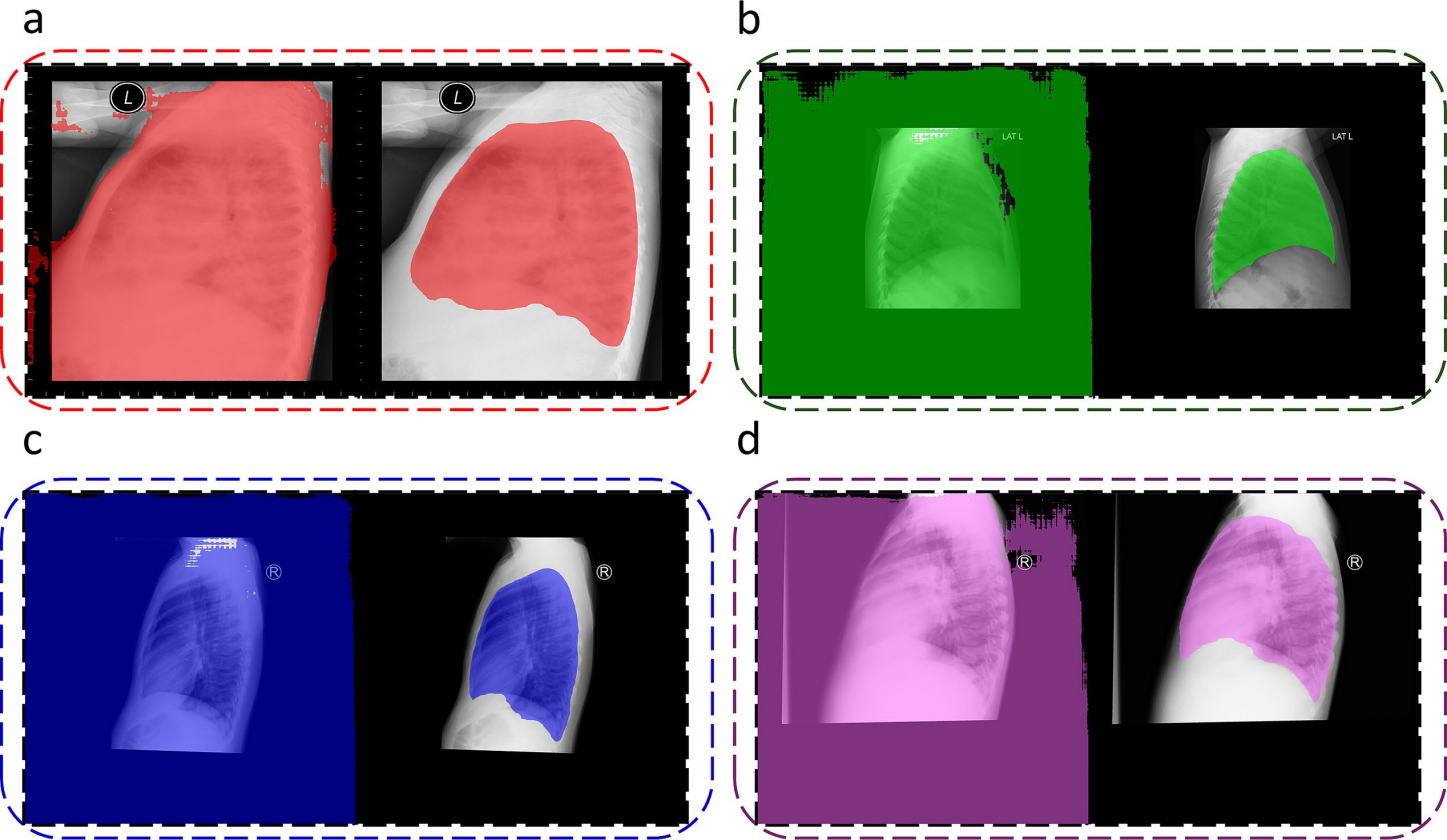
Representative comparisons of MedSAM segmentation using non-targeted (left) vs. targeted (right) prompting across pediatric age groups. Targeted segmentation uses YOLO11s-predicted bounding boxes as spatial priors, demonstrating notably improved precision in lateral lung field segmentation. **(a)** 0 years to < 2 years (red); **(b)** 2 years to < 5 years (green); **(c)** 5 years to < 11 years (blue); **(d)** 11 years to < 18 years (cyan).

The segmentation analysis presented in this section is intended solely as a demonstration of the benefit of spatially targeted prompting as a downstream application of PLUTO’s detection output. It does not constitute a segmentation benchmark; rather, it illustrates a well-recognized principle in medical imaging pipeline design, that accurate spatial localization meaningfully improves the quality of subsequent segmentation (Litjens et al., 2017). A critical yet often underappreciated step in building reliable medical imaging pipelines is the role of spatial localization before segmentation (Litjens et al., 2017). We investigated the influence of lung field detection as a guiding prior in downstream segmentation using MedSAM under ZSL settings. Specifically, we compared two prompting strategies: (1) non-targeted segmentation, where the entire image served as the bounding box input to MedSAM, and (2) targeted segmentation, where the lung field bounding box predicted by the YOLO11s model was used to spatially guide the segmentation process. As shown in Figure 8, the performance of MedSAM with targeted prompts consistently outperformed its non-targeted counterpart across all pediatric age groups. Non-targeted segmentation (left panels) over-segmented irrelevant regions and introduced noise due to a lack of spatial constraint. In contrast, when guided by bounding box predictions (right panels), the segmentation output demonstrated markedly refined contours that were restricted to a spatially meaningful region. This observation reinforces the importance of RoI detection as a critical precursor in segmentation workflows. Detection-based prompting focuses segmentation models on relevant structures, reducing spurious predictions and enhancing boundary precision. This is especially beneficial in pediatric lateral CXR analysis, where complex overlapping anatomy and variability in lung morphology across the age range could compromise downstream segmentation, disease detection and classification, and clinical decision support quality and performance.

### 6.5 Clinical expert evaluation of detection-guided segmentation

**Figure 9:**
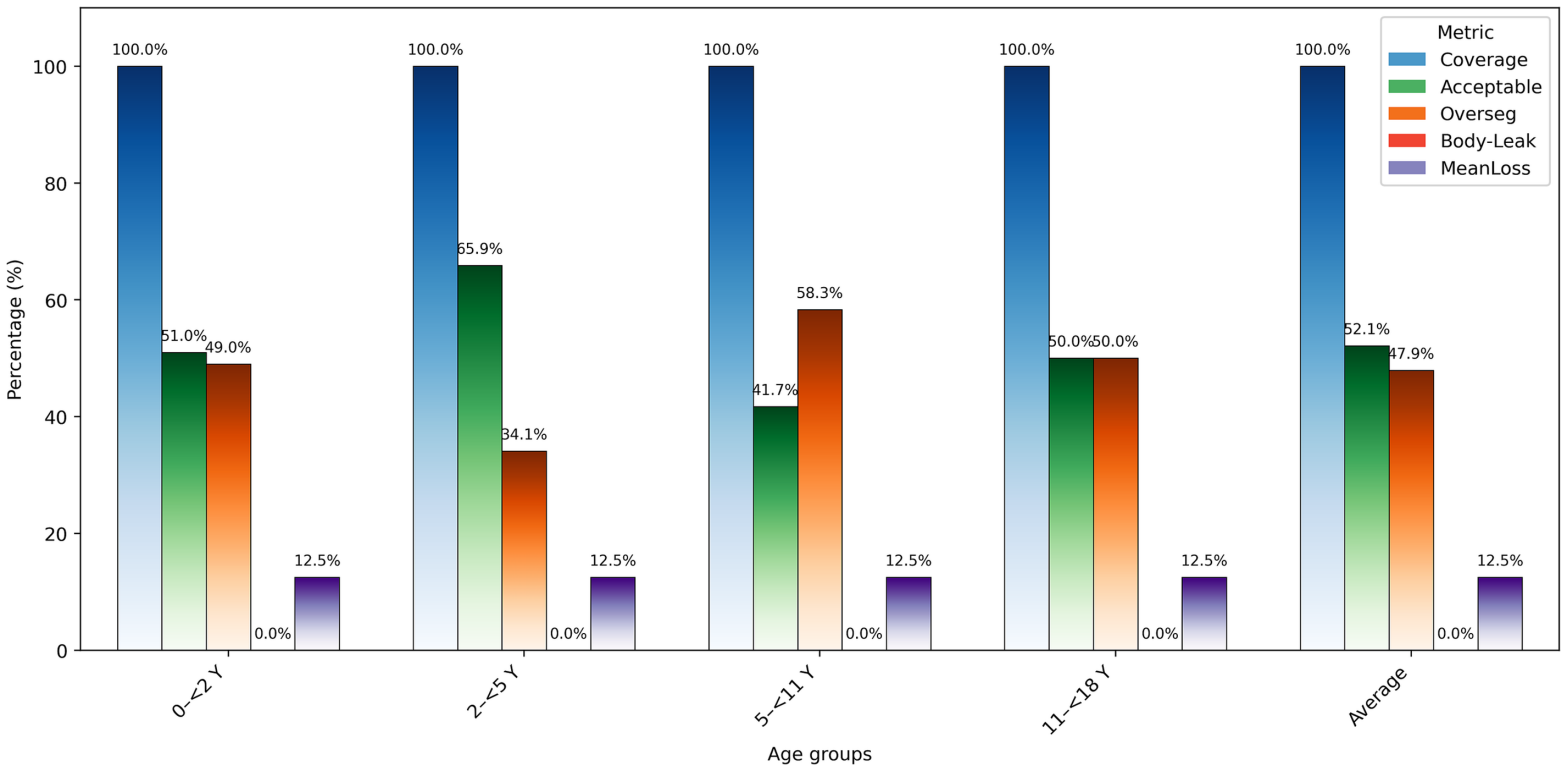
Grouped bar plot showing percentage coverage, acceptable segmentation, over-segmentation, Body-leak rate, and mean under-coverage loss by pediatric age group and mean overall percentage (Y = years).

We designed a qualitative evaluation for the clinical expert to assess the validity of bounding-box-driven zero-shot lateral lung field segmentation. The document displayed the pediatric test CXR, both from internal and external sets, with and without the corresponding zero-shot segmentation output mask generated by the MedSAM model. The segmentation mask was overlaid on the original image to facilitate an intuitive visual comparison. Each image was labeled with its pediatric age group to ensure age-specific evaluation. Figure 9 provides a visualization of the aggregated responses of the expert’s evaluation using four questions that were aimed at capturing the segmentation quality and anatomical relevance. The evaluation criteria included, (1) whether the segmentation mask successfully covered the lung field (Coverage metric in Figure 9), (2) if adequate lung field coverage was achieved (Acceptable), (3) assessment of any over-segmentation resulting in segmented regions extending outside chest wall (Overseg, and Body-Leak), and (4) assessment of under-segmentation that attempted to approximately quantify the loss into 0–25%, 25–50%, 50–75%, or 75–100%, respectively, (MeanLoss). The assessment revealed that MedSAM achieved 100% lung field coverage across all pediatric age groups following bounding-box guidance. Despite variations in segmentation quality, between 41.7 and 65.9% of segmentations (mean: 52.1%) were deemed fully acceptable and without extraneous regions. Importantly, any over-segmentation was within the chest wall. For all cases of under segmentation, the missed area was less than 25% of the lung field. The results emphasize the reliability induced by a RoI detector on zero-shot, fully automatic segmentation.

### 6.6 Limitations

Our study introduces a comprehensive DL-based framework for age-independent lung field detection in lateral-view chest radiographs, with a particular emphasis on pediatric imaging, a domain that remains critically underexplored in radiographic AI research. By systematically evaluating multiple YOLO11 variants, we demonstrate that our YOLO11s-based PLUTO model achieves consistently strong performance on pediatric CXRs while also demonstrating consistent generalizability to adult datasets. We are releasing the PLUTO model weights to support transparency, reproducibility, and further methodological development in this space.

Despite the contributions of this work, several limitations warrant acknowledgment. The dataset size, particularly when stratified by pediatric age groups, remains modest. This constraint limits the development of age-specific foundational models and underscores a broader challenge in pediatric CXR research: the scarcity of high-quality, well-annotated pediatric imaging data essential for advancing automated tools for cardiopulmonary disease conditions. Three dataset-related constraints are especially relevant. The adult generalizability evaluation used 100 images per adult cohort (PadChest and IU CXR), yielding encouraging but not comprehensive cross-domain evidence. The 11 to <18 years pediatric subgroup in the NIAID pediatric-CXR dataset comprised only nine cases, reflecting real-world acquisition patterns in pediatric lateral imaging rather than a study design choice; age-stratified conclusions for adolescents should therefore be regarded as descriptive and qualitative. External pediatric validation similarly relied on 23 PadChest lateral CXRs, the largest publicly available source at the time, which does not constitute a sufficiently powered external benchmark; the scarcity of annotated pediatric lateral CXR collections is a key motivation for our open release of model weights. Finally, the current scope of PLUTO is intentionally restricted to detecting the lung field in lateral projections as a necessary foundational step and does not yet extend to finer-grained anatomical structures or disease-specific radiographic markers. Future work should explore multi-task detection frameworks capable of jointly identifying cardiopulmonary structures and pathology-relevant features to better support downstream diagnostic applications. Annotation reliability is a further methodological consideration. Bounding boxes were produced by a single annotator and verified by one clinical expert, and formal inter-rater reliability statistics were not computed. Future studies should incorporate multiple independent annotators with quantitative agreement metrics to more rigorously characterize annotation consistency. The zero-shot MedSAM segmentation analysis was conducted in the absence of pixel-level ground truth masks, as the study’s annotation protocol was designed exclusively for bounding box detection. Standard quantitative segmentation metrics could not therefore be computed against a reference standard. This analysis should be interpreted as a proof-of-concept demonstration of detection-guided segmentation improvement rather than a segmentation performance benchmark. Acquiring expert pixel-level segmentation annotations and conducting a rigorous quantitative segmentation evaluation represents an important and clearly defined direction for future work.

## 7 Conclusion

In summary, PLUTO provides bounding-box-based lateral lung field localization in pediatric CXRs and demonstrates preliminary cross-domain generalizability to adult images. It is important to state explicitly that PLUTO does not detect or diagnose TB, lymphadenopathy, or other thoracic pathologies; it provides a spatial prior, a necessary anatomical foundation, upon which downstream diagnostic AI models can be built and evaluated. Clinical translation will require the subsequent development and validation of complete AI-assisted diagnostic workflows that incorporate PLUTO’s detection output as a first stage. With this scope clearly defined, this work establishes a methodological foundation for automated pediatric lateral CXR analysis and provides openly available model weights to support the research community. Continued progress will require larger and more diverse pediatric lateral CXR datasets, multi-annotator annotation frameworks, and the development of multi-structure, pathology-aware detection models capable of jointly localizing cardiopulmonary anatomy and pathology-relevant radiographic features.

## Data Availability

The NIAID pediatric-CXR data cannot be made publicly available as sharing is controlled by strict data transfer agreements with respective data providers. The PadChest dataset is publicly available and can be accessed from https://bimcv.cipf.es/bimcv-projects/padchest. Similarly, the Indiana University CXRs and associated reports are publicly available through the Open Access Biomedical Image Search Engine (Open-i^®^) at https://openi.nlm.nih.gov/faq?download=true. Code used in this study can be found at https://github.com/antani-lab/YOLO-for-lateral-lung-detection-SRK. No protected health information or personally identifiable information is included.
