# Correction: PLUTO: a YOLO-based lung field detector for pediatric lateral chest X-rays generalizable to adults

**DOI:** 10.3389/frai.2026.1944599

**Published:** 2026-08-03

**Authors:** Sivaramakrishnan Rajaraman, Renee Browning, Patrick Jean-Philippe, Carlos M. Perez-Velez, Sameer Antani

**Affiliations:** 1 Division of Intramural Research, National Library of Medicine, National Institutes of Health, Bethesda, MD, United States; 2 National Institute of Allergy and Infectious Diseases, National Institutes of Health, Bethesda, MD, United States; 3 Division of Infectious Diseases, School of Medicine, University of New Mexico, Albuquerque, NM, United States

**Keywords:** adult generalizability, deep learning, lateral chest X-rays, lung field detection, pediatric, YOLO11 object detection

Authors Carlos M. Perez-Velez and Sameer Antani were erroneously omitted as equal senior contributing authors.

The original version of this article has been updated.

